# Telomere Tales: Exploring the Impact of Stress, Sociality, and Exercise on Dogs’ Cellular Aging

**DOI:** 10.3390/vetsci12050491

**Published:** 2025-05-19

**Authors:** Luisa Mascarenhas Ladeia Dutra, Flaviane S. Souza, Angelica Silva Vasconcellos, Robert J. Young, Ivana Gabriela Schork

**Affiliations:** 1 School of Science, Engineering & Environment, University of Salford, Manchester M5 4WT, UK; flaviane@ufrb.edu.br (F.S.S.); r.j.young@salford.ac.uk (R.J.Y.); 2 Instituto de Educação Continuada (IEC), Pontifícia Universidade Católica de Minas Gerais, Belo Horizonte 30535-901, Brazil; 3 Centro de Ciências da Saúde, Universidade Federal do Recôncavo da Bahia, Cruz das Almas 44380-000, Brazil; 4 Programa de Pós-Graduacão em Biodiversidade e Meio Ambiente, Pontifícia Universidade Católica de Minas Gerais, Belo Horizonte 30535-901, Brazil; angelicavasconcellos@pucminas.br; 5 Department of Animal and Agriculture, Hartpury University, Gloucester GL19 3BE, UK; ivana.schork@hartpury.ac.uk

**Keywords:** chronic stress, cumulative experiences, aging, animal welfare

## Abstract

Animal welfare is influenced by the cumulative life experiences of an individual. Among these, exposure to chronic stressors has a significant impact on both physical and mental health, contributing to premature aging—a process linked to telomere shortening. Conversely, positive experiences have been shown to mitigate, delay, and sometimes reverse telomere attrition. This suggests that telomere length could be a reliable indicator for assessing animal welfare. This study explored the association between telomere length and characteristics such as life history, environment, and health in domestic dogs. Buccal swabs collected DNA samples from 250 dogs, and telomere length was quantified via qPCR. Our findings revealed that environmental factors significantly influenced telomere length. Dogs housed in kennels or subjected to low physical activity levels exhibited shorter telomeres. Similarly, dogs living in groups of more than five dogs had shorter telomeres, and male dogs were found to have longer telomeres than females. Overall, these results highlight the importance of environmental conditions in influencing telomere length in dogs and the potential to use this biological indicator to evaluate animal welfare.

## Simple Summary

Dogs, like humans, experience aging, which is influenced by their environment and life experiences. This study explored how different living conditions and life histories affect the aging process in dogs by examining their telomeres—structures located at the ends of chromosomes that preserve the structural integrity of DNA and, therefore, prevent degradation associated with cellular senescence. Shorter telomeres are linked to stress and aging. Through a non-invasive method to collect DNA from dogs, our study investigated the relationship between telomere length and dogs’ backgrounds. We found that dogs living in varied environments, such as family homes, kennels, or working as police dogs, showed different rates of telomere shortening. Interestingly, while factors like age and breed did not strongly influence aging, dogs in stressful or less enriching conditions, such as laboratories, had the shortest telomeres. In contrast, working dogs often had longer telomeres, possibly due to structured environments and meaningful activities. These findings help us understand how a dog’s life circumstances affect its health and aging, providing valuable insights for improving animal care, health monitoring, and welfare policies. Considering positive and negative experiences, this research could also guide how we support aging in pets and working animals, ensuring healthier lives.

## 1. Introduction

Dogs (*Canis familiaris*) are not only valued as companions but also for their ability to work closely with humans, a role facilitated by their social nature and trainability [1]. They contribute significantly to various domains such as farming, hunting, guarding, military operations, search and rescue missions, and therapy. However, the demands associated with these roles can negatively impact their welfare [2,3]. For instance, police and military dogs often face acute stress during their duties, whereas laboratory dogs are more prone to chronic stress [4]. Similarly, even pet dogs can experience considerable differences in welfare depending on the household environment, quality of care, and ownership practices [5].

Assessing animal welfare requires tailoring methods to individuals’ specific circumstances and environments. However, dogs’ diverse lifestyles complicate the evaluation of their well-being. Moreover, many physiological indicators, such as stress hormone levels, reflect stress responses from the past hours or days, offering limited insight into experiences that occurred years earlier but may have significantly influenced the individual’s overall quality of life [6].

Telomeres are segments of DNA that protect the ends of chromosomes, gradually shortening with each cellular division. This progressive reduction contributes to replicative senescence or cell death through apoptosis [7]. Factors such as an unhealthy lifestyle (e.g., poor diet) [8] or environmental stressors (e.g., chronic stress) [8] can accelerate telomere shortening. Research on species such as macaques [9], domestic dogs [10], and birds [11] has demonstrated a link between telomere attrition and stress. Evidence suggests that positive experiences may mitigate or even reverse telomere attrition, indicating that telomere length could provide insights into an animal’s cumulative life experiences and overall welfare status, whether positive or negative [12,13,14,15].

Since stress is a key parameter in animal welfare assessments, telomere shortening is proposed as a potential welfare indicator [12]. Previous studies have explored the relationship between telomere shortening and the most common method of stress assessment, cortisol levels. Some report significant associations between telomere shortening and serum or hair cortisol, while others have found mixed or inconclusive results [12,13]. These discrepancies may be due to species-specific differences, variations in stress measurement methods, or the influence of additional confounding factors.

Research on telomere attrition in dogs has primarily focused on understanding the relationship between telomere length, mortality, lifespan, and the activity of telomerase, an enzyme that preserves telomere integrity in dog tissues [10,14]. Findings suggest that smaller dog breeds have longer telomeres, correlating with their extended lifespans, while larger breeds tend to age faster and have shorter lifespans due to their shorter telomeres [10,14]. Additionally, telomeres play a pivotal role in regulating the lifespan of somatic cells [15]. However, to date, no studies have specifically investigated the connection between telomere attrition and dog welfare.

This study presents the development and validation of a non-invasive method to assess the quality of life in dogs by measuring relative telomere length (rTL) and examining its association with the dogs’ background.

## 2. Materials and Methods

### 2.1. Ethical Statement

This study was conducted following ethical guidelines and was approved by the relevant ethics committees in Brazil (process no. 0056/2017) and the United Kingdom from University of Salford Ethics Committee (approval number STR1617-22). Biological materials were collected under DEFRA license ITIMP16.1096. No additional ethical approval was required for sample collection at this facility. All institutions and organizations will remain anonymous in accordance with GDPR guidelines and the agreement between the University of Salford and the third parties involved.

### 2.2. Subjects and Life History Questionnaire

This study included 250 domestic dogs from diverse backgrounds for telomere length assessment. The dogs’ DNA samples were collected from various locations in Brazil and Europe (Table 1). The dogs were classified by sex, age, and background, which was divided into five categories: companion animals, shelter dogs, working dogs, laboratory dogs, and rehomed individuals (Table 1).

To limit confounding factors that could compromise the interpretation of our results, we conducted a prescreening selection of dogs. To the best of our knowledge, none of the dogs in the study had long-term chronic conditions or exhibited any recurrent behavioral issues that could affect their overall well-being.

To explore the potential impact of the dogs’ environment and life history on their telomere length, a detailed questionnaire was administered to gather information on multiple aspects of each dog’s life. Respondents provided details regarding (i) breed, (ii) previous ownership (whether the dog was with its first owner or rehomed), (iii) neutering status, (iv) overall health condition, (v) training background, (vi) dietary habits, (vii) sleeping site, (viii) exercise frequency, and (ix) social interactions with both humans and other animals. Some of these variables were not included in the final analysis due to data completeness constraints and to maintain model clarity. Shelter dogs were group-housed in large enclosures outside, and the duration of stay in the shelter varied individually. Research dogs were group-housed in 900 m^2^ enclosures outside. Shelter dogs had a doghouse available in every enclosure that could fit all the pack, keeping them safe from any weather conditions. Laboratory dogs were pair-housed under standardized care conditions, following ethical and welfare guidelines.

The breed of each dog was determined based on the UK Kennel Club criteria [16], with dogs of unknown breed categorized as “Mix”. According to these criteria, dogs were grouped into categories: Gundogs, Hounds, Pastorals, Terriers, Toys, Utility, Work, and Mix.

An additional categorization was used to differentiate dogs that fell under the working dog category. For these dogs, animals were grouped based on their work location, either UK or Brazilian working dogs. However, this was not intended solely to reflect the geographical location of the dogs, but rather to highlight the distinct characteristics of this type of activity in each country, such as training and management. The Supplementary Material (Supplementary Tables S1–S3) provides a comprehensive list of individual characteristics for all 250 dogs and the options provided in the questionnaire.

### 2.3. DNA Sampling and Telomere Length Measurement

Saliva and tissue samples to extract DNA were collected using buccal swabs. The swab was gently rubbed against the inner cheek of the dog’s mouth for a few seconds to extract the material. The procedure was carried out by either a veterinarian, the owner, or the caretaker to minimize the animal’s stress. Positive reinforcement was used during the procedure to encourage the dog’s cooperation.

Genomic DNA was isolated from the buccal cell samples using the Buccalyse DNA Release Kit, following the manufacturer’s guidelines. The concentration of the extracted DNA was then quantified with the NanoDrop^®^ ND-1000 UV-Vis Spectrophotometer (Thermo Fisher Scientific, Waltham, MA, USA). We set a target DNA concentration of 100 ng/μL as the standard for inclusion in the reactions. Any samples with concentrations higher than this threshold were diluted to ensure minimal variation across samples, helping maintain consistency in DNA concentration throughout the analysis.

Quantitative PCR (qPCR) was conducted to assess telomere length. Each qPCR run incorporated two components: (i) a tenfold serial dilution derived from a pooled DNA sample of 250 dogs at a concentration of 100.7 ng/µL, which was used to generate the standard curve and optimize the primers; (ii) no-template controls (NTC) to identify potential contamination and primer dimer formation [17]. To minimize plate-to-plate variation, all reactions were performed under identical conditions using the same reagent batches, primer sets, and instrument settings. Although inter-run calibrators (IRCs) were not used in this study, standard curve normalization and technical replicates (each sample run in triplicate) were employed to ensure consistency across runs [18]. The primers employed in this study consisted of telomere-specific primers, telg (5′-ACACTAAGGTTTGGGTTTGGGTTTGGGTTTGGGTTAGTGT-3′) and telc (5′-TGTTAGGTATCCCTATCCCTATCCCTATCCCTATCCCTAACA-3′), as described in [19]. Additionally, 18S primers were used for the reference gene, including 18S sense (5′-GAGGTGAAATTCTTGGACCGG-3′) and 18S antisense (5′-CGAACCTCCGACTTTCGTTCT-3′), from a previously established study [20]. Primer performance in qPCR reactions was assessed using Rotor-Gene Q Series software (version 2.3.1), with efficiency values ranging between 98% and 100%.

In this research, we utilized a monochrome multiplex qPCR assay, adapting the approach described by [18]. The master mix reactions followed the protocol established by [21], and amplifications were conducted on a Rotor-Gene^®^ Q cycler (QIAGEN) equipped with Rotor-Gene Q software (version 2.3.1), using 0.1 mL strip tubes and caps. The Rotor-Gene Q Series software created a standard curve, considering the dilution factors for telomere and reference genes in each sample. The master mix preparation for the qPCR reaction followed the protocol established by [19] and validated for dog samples by [21]. Each reaction mixture contained 10 µL of Power SYBR Green master mix (2×) at a final concentration of 1×, as well as 1 µL of telomere-forward primer (2 mM) and 1 µL of telomere-reverse primer (2 mM), both at a final concentration of 0.1 mM. Additionally, 4 µL of H_2_O was included to adjust the reaction volume, along with 4 µL of DNA (5 ng/µL), resulting in a total of 20 ng of DNA per reaction. The qPCR reaction mixture and conditions consisted of an initial denaturation step at 95 °C for 15 min, followed by two cycles of 94 °C for 15 s and 49 °C for 15 s. This was succeeded by 40 amplification cycles at 94 °C for 15 s, 62 °C for 10 s, and 74 °C for 15 s with signal acquisition. Additional steps included 84 °C for 10 s and 88 °C for 15 s, both with signal acquisition. The reaction concluded with a melting curve analysis, incrementally increasing the temperature from 72 °C to 95 °C in 0.5 °C steps every 30 s. Each run incorporated a standard curve generated by the Rotor-Gene Q Series software (version 2.3.1), which accounted for the dilution factors corresponding to the telomere and reference gene quantities for each sample [21].

To measure the relative telomere length, we employed a modified qPCR based on the method described by [22] and utilized a multi-copy gene as the reference instead of a single-copy gene, as validated by [23]. This calculation was performed by determining the ratio of the telomere repeat copy number (T) to the reference gene copy number (S). Each sample was analyzed in duplicate, yielding two T/S values. In cases where discrepancies were observed between the duplicates, a second assay was performed. The final measurement for each sample within a given run was obtained by averaging the duplicate values [22].

### 2.4. Statistical Analyses

Statistical analyses were conducted using linear discriminant analysis (LDA) to verify whether the grouping of dogs based on their backgrounds (i.e., pet, shelter, work, laboratory, or rehomed dogs) and age was appropriate for subsequent analysis. The LDA required a minimum of two samples per category, leading to the exclusion of a 14-year-old dog from the UK work group, as it did not meet this requirement.

The relationships between distinct characteristics of dogs’ lives and telomere length were analyzed using generalized linear models. A normality test indicated that RTL did not meet the normality assumptions (Shapiro–Wilk test result, W = 0.96327, and a *p*-value < 0.001). Additional tests on the skewness data produced a value of 0.68, suggesting a right-skewed distribution. Based on this, a Gamma distribution with a log link was selected as the family for analysis; the log link function helps stabilize variance and leads to more homoscedastic residuals in the model, thereby enhancing the model’s performance.

Model selection was based on Akaike’s information criterion (AIC), allowing the identification of the most explanatory model. ANOVA was used as a post-hoc test to examine differences in rTL across multiple categories. The initial model included data from 250 sampled dogs to explore how various factors influenced rTL. The variables assessed were sex, age, breed group, origin, neuter status, overall health, training, frequency of food treats, sleep location (housing), exercise, interaction with people, and interaction with other animals.

A subsequent analysis assessed potential differences between police work dogs from Brazil and the UK. Although these dogs shared the same occupation, differences in kenneling conditions and workload could significantly affect rTL. As a result, a second model was designed to assess how the country (Brazil or the UK), work schedule, and job type influenced the rTL of 65 police working dogs. Similar to the first model, rTL was the response variable. Additional explanatory variables included in the full model were sex, breed, category group, neutered status, and health status.

Data normality and the best fit for GLM models were analyzed using RStudio Desktop version 2025.05.0+496 (RStudio Team, 2020) with the “MASS” [24], “car” [25], and “AICcmodavg” [26] packages. A significance level of *p* < 0.05 was used.

## 3. Results

### 3.1. Association of Telomere Length and Age

A total of 249 dogs were included in the analysis of relative telomere length. The overall mean rTL was 0.744 (SD = 0.080), with values ranging from 0.550 to 0.990. The median rTL was 0.730, with an interquartile range (IQR) from 0.690 to 0.780 (Table 2).

A discriminant analysis was conducted using rTL as a predictor to assess the appropriateness of grouping dogs by age. The results revealed that the accuracy of age group assignment was 25.33%, with younger dogs being more frequently misclassified into incorrect age groups, while older dogs were more often correctly assigned to their actual age category.

When analyzing the mean rTL across the different age groups, it was observed that the mean rTL exhibited less variation in younger dogs than in adults and senior dogs. The standard deviation (SD) in younger dogs was also lower, maintaining this trend (Table 2).

### 3.2. Model 1: Factors Impacting a Dogs Relative Telomere Length

A generalized linear model (GLM) with a Gamma distribution and log link function was constructed to verify the relationship of several predictors with RT Length. The best-fitting model found that category, sex, sleep location, walking frequency, and contact with other animals all affected telomere length. The model demonstrated a pseudo-R^2^ (Cox and Snell) of approximately 0.313, indicating that about 31.3% of the variance in RTL was explained by this model. A significant effect was observed: F(14, 232) = 4.68, *p* < 0.0001, η^2^ = 0.0082. A summary of the GLM results is presented in Table 3.

**Figure 1:**
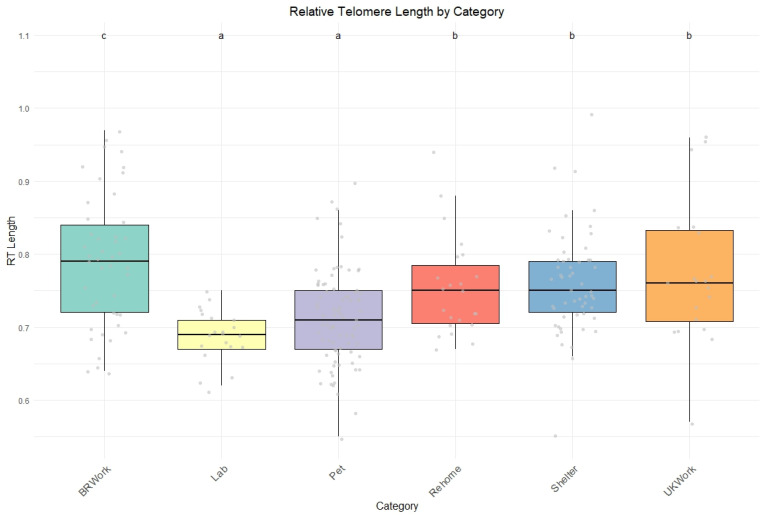
Relative telomere length in the sampled dogs (N = 250) based on their background category. Different colors represent different dogs’ groups (green = BR work, yellow = laboratory, purple = pet, red= rehomed, blue = shelter, orange = UK work). Gray circles indicate the dispersion of data points. Boxes represent interquartile ranges, tick lines represent the median, and whiskers show maximum and minimum values. Different letters indicate statistical significance at *p* < 0.05.

The dogs’ category based on their origin was a strong predictor of telomere length, with laboratory dogs (β = −0.1737 ± 0.0448, *p* = 0.0001), followed by pet dogs (β = −0.1277 ± 0.0426, *p* = 0.0030), having, on average, shorter telomeres than other groups (Figure 1).

**Figure 2:**
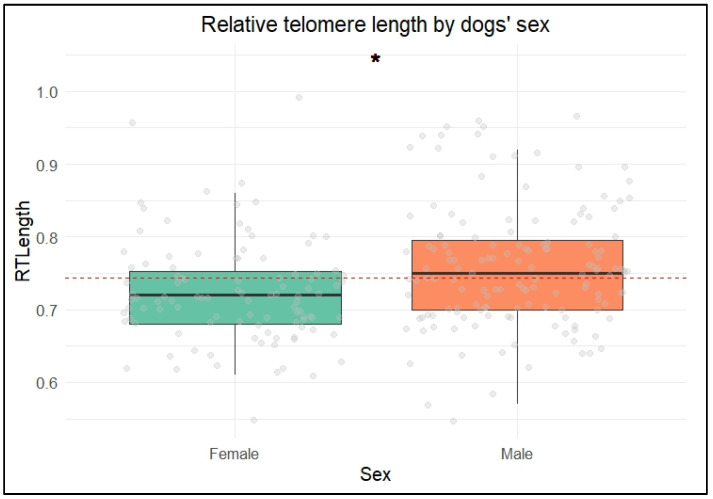
Differences in relative telomere length between male and female dogs. Different colors represent different sexes (green = female, orange = male). Gray circles indicate the dispersion of data points. Boxes represent interquartile ranges, tick lines represent the median, and whiskers show maximum and minimum values. The star indicates statistical significance at *p* < 0.05.

Male dogs had significantly higher rTL than females (β = 0.02 ± 0.0124, *p* = 0.03) (Figure 2).

In relation to different management practices, dogs that were exercised every other day showed a significant increase in telomere length (β = 0.13 ± 0.02, *p* ≤ 0.01) compared to dogs that received different types of exercise. Dogs that slept outside in a kennel had a significantly shorter rTL than those sleeping elsewhere (β = −0.09 ± 0.03, *p* = 0.01). Likewise, contact with other animals was also negatively associated with rTL, with dogs having contact with one to two animals (β = −0.06 ± 0.02, *p* ≤ 0.01) and those in contact with morethan five animals (β = −0.07 ± 0.0294, *p* = 0.01) showing a significant reduction in telomere length. Other factors, such as sleeping in the owner’s bed, contact with three to four animals, and certain categories (rehomed, shelter, UK work), were not significant predictors of rTL (*p* > 0.05).

### 3.3. Model 2: Relative Telomere Length in Relation to Dogs’ Work Type

A total of 65 dogs were included in this analysis. The overall mean rTL was 0.784 (SD = 0.093, range = 0.570–0.970). The interquartile range (IQR) was 0.120, and the coefficient of variation (CV) was 11.9%, indicating moderate relative dispersion in rTL values (Table 4).

When comparing geographic groups, individuals from Brazil (N = 45) had a higher mean rTL (0.790 ± 0.091, CV = 11.57%) than those from the UK (N = 20, mean 0.772 ± 0.098, CV = 12.72%). RTLength also varied by working type; the drug/gun category (N = 14) exhibited the highest mean RTLength (0.828 ± 0.084, IQR = 0.135, CV = 10.16%), whereas search and rescue (N = 7) had the lowest mean RTLength (0.693 ± 0.042, CV = 6.11%) (Table 4).

A subsequent GLM test was conducted to investigate the background factors that may affect relative telomere length (rTL) in police working dogs from Brazil and the UK. Although both groups shared the same occupation, they had differences in their environments and routines. These included variations in the walk routine, sleeping site, food reinforcement (treats), and training style, which were consistent within each group but differed between the two. The GLM test returned a significant effect (F(8, 56) = 4.16, *p* < 0.0001, η^2^ = 0.0062), and the overall model demonstrated a pseudo-R^2^ (Cox and Snell) of approximately 0.1963, indicating that nearly 20% of the variance in the relative telomere length was explained by the model. The results for this model, including the effects of these factors on rTL, are summarized in Table 5.

The results showed marginally significant differences between UK and Brazilian dogs (β = −0.0511 ± 0.0262, *p* = 0.0561), suggesting that work-type location does have a weak effect on telomere length.

**Figure 3:**
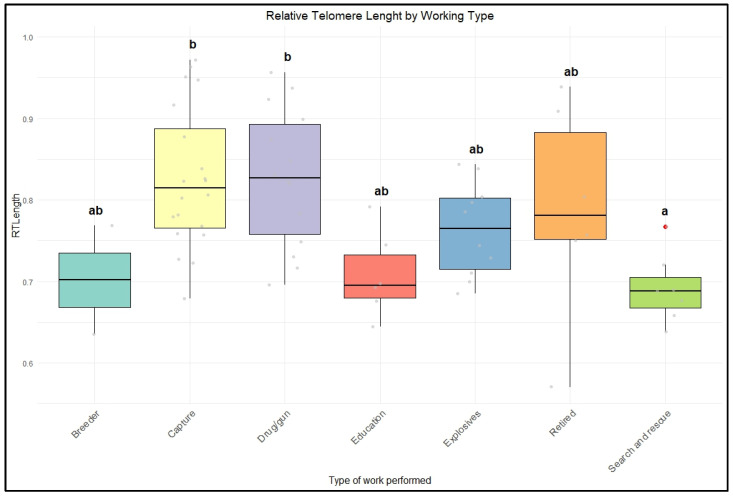
Relative telomere length (rTL) differences between police dogs based on the type of work the individuals perform. Different colors represent different types of work (emerald green = breeder, yellow = capture, purple = drugs and guns, red= education, blue = explosives, orange = retired, and lime green = search and rescue). The gray circles indicate the dispersion of data points, and the red circles indicate outliers. Boxes represent interquartile ranges, tick lines represent the median, and whiskers show maximum and minimum values. Different letters indicate statistical significance at *p* < 0.05.

Additionally, the type of work performed by the dogs was associated with differences in rTL. Dogs engaged in tracking (β = 0.1730 ± 0.0833, *p* = 0.0425) and those working with drugs and guns (β = 0.1606 ± 0.0829, *p* = 0.0579) had longer relative telomere lengths than those involved in other types of work (Figure 3). These findings suggest that geographic location and the specific nature of a dog’s work can significantly impact their rTL.

## 4. Discussion

This research has highlighted the complex interactions among dogs’ backgrounds, environments, and husbandry practices that influence their relative telomere length (rTL). While previous studies have primarily focused on individual factors, such as age, social isolation, or reproduction, this study takes a broader approach by examining multiple factors and their cumulative effects on rTL.

While age is generally expected to correlate with telomere length, our results did not meet these assumptions. Our findings reveal that dogs with similar routines and social interactions tend to exhibit comparable rates of cellular senescence, as indicated by their rTL. However, the discriminant analysis still identified some inconsistencies among dogs’ classification, which suggests that other characteristics may play a significant role in determining telomere attrition, reinforcing that the dog’s life experiences may impact their biological aging process.

Based on this premise, our study found that laboratory dogs exhibited the lowest relative telomere lengths (rTLs) compared to the other groups. This finding supports previous research, which highlights that dogs in laboratory environments often experience a poorer quality of life due to suboptimal housing conditions, inadequate handling, and social isolation [27]. These adverse conditions are associated with higher stress responses, as reflected in increased cortisol levels, and often lead to the development of stereotypic behaviors [28,29]. Previous studies have reported mixed findings regarding the relationship between telomere shortening and cortisol levels, with some identifying significant associations [30,31], while others remain inconclusive [30]. Early life experiences, including shelter histories, may significantly influence the behavior and biological aging of dogs, as prior studies have shown that such factors can impact welfare and development [32]. Our results add to the growing body of evidence suggesting that environmental and social stressors in laboratory settings can accelerate biological aging, as indicated by the shortened telomeres in these dogs.

Pet dogs also have shorter telomeres than other groups. Among different dog groups, pets are perceived as having a better quality of life, largely due to their unique relationship with humans and the dedicated healthcare they receive. However, pet dogs may face significant challenges due to their living conditions, such as a lack of physical activity, overfeeding, or poor socialization management [32,33], which can lead to chronic stress [32,34] and even impact their lifespan [35]. For instance, our results suggest that increased exercise has a positive impact on telomere length. In contrast, contact with other animals may affect relative telomere length, further supporting the evidence for the association of lifestyle factors in pet dogs and their impact on health and welfare. Furthermore, other factors, such as mental stimulation through training, have been shown to influence telomere attrition [36,37] and have also demonstrated variability in telomere lengths across dog breeds, correlating with differences in lifespan. If pet ownership trends favor specific traits and management styles, this could directly contribute to the observed reduction in telomere length among pet dogs.

Despite our results not finding a specific association with breed and training, this is most likely due to the heterogeneous nature of the sample and the grouping of breeds by broader types. Therefore, further studies could focus on exploring the various factors present in pets’ lives to better understand the impact that ownership choices may have on dogs’ aging.

Across the groups, males exhibited longer relative telomere lengths (rTLs) than females. This finding contrasts with studies conducted in rats and humans, which have typically found that females have longer telomeres than males. In mammals, the longer lifespan of females is often attributed to having two X chromosomes, which enhances DNA repair mechanisms [38,39,40]. The unexpected result in dogs may suggest that the factors influencing rTL in dogs are different from those observed in other mammals, potentially reflecting species-specific biological mechanisms or the influence of environmental and management aspects [10,41]. Furthermore, we did not find any significant association between rTL and reproductive status, despite the existing body of evidence on the relationship between hormone levels and neutering, which could potentially interfere with dogs’ health. More than 50% of our sample consisted of entire dogs, which could explain the result; however, further research could support understanding whether changes in the hormone profile after sterilization contribute to telomere attrition.

In our study, the location where pet dogs slept was associated with telomere length, with dogs that slept in kennels having shorter telomeres than those allowed inside the house or on their owner’s bed. While research on dog sleeping arrangements is scarce, some studies have suggested that co-sleeping with owners may contribute to better animal welfare. Previous research [42] has found that co-sleeping could reduce anxiety in dogs, although they noted that more comprehensive data across a dog’s lifetime is necessary to understand its effects fully.

Outdoor kenneled dogs may face negative environmental factors, such as harsh weather, loud noises, and discomfort. These conditions can induce physiological stress, leading to increased oxidative stress. Studies in other species have linked environmental metrics with elevated stress responses and telomere attrition (see review [43]).

Moreover, sleep quality and consistency may be compromised in kennel environments, further exacerbating stress responses. Research with laboratory dogs has highlighted the association between sleep, stress, and environmental metrics, such as noise and temperature [44]. Given that sleep quality is associated with telomere length maintenance [45], outdoor sleeping conditions may indirectly contribute to accelerated cellular aging.

In this study, dogs that were walked every day had shorter relative telomere lengths (rTLs) than dogs that were never walked or walked every other day. This finding was initially unexpected, as walking is often considered a key element of responsible dog ownership and necessary to ensure a dog’s welfare [46]. However, a possible explanation for this result lies in the walks’ dynamics.

For instance, walking equipment, such as leashes and harnesses, might cause discomfort or restrict the dog’s ability to move freely. This restriction could generate stress, impacting telomere length [47]. Furthermore, daily walks may be brief, with the primary purpose being functional (e.g., to fulfill the dog’s physiological needs) rather than providing opportunities for more enriching experiences, such as exercise, socialization, or mental stimulation. Researchers [48] suggested that some dog owners might take their dogs on short walks to avoid feeling guilty about not providing exercise without considering that longer walks could offer additional benefits. Short walks, particularly when they do not meet the dog’s need for physical and mental stimulation, can lead to frustration and stress, factors commonly associated with higher cortisol levels. Over time, this chronic stress could accelerate telomere attrition.

Additionally, walking is often used to mitigate behavioral issues in dogs. However, if the owner is not adequately advised by a professional, daily walks could expose the dog to stressors that, when chronic, may contribute to adverse health outcomes, including impacts on rTL [45,47,49].

Since this study focused on background factors rather than specific activity patterns, follow-up questions regarding the duration of daily walks, the dog’s emotions during the walk, or the type of walking equipment used were not included in the questionnaire. In dogs that are reactive to other dogs on walks, this activity tends to have a more negative effect because it stresses them, and their owners are also stressed—a double hit on dog welfare [50]. Thus, dog walks may include both negative and positive aspects, and for some dogs, the negatives might outweigh the positives; therefore, further investigations are necessary to better understand the relationship between walking habits and telomere length.

Our study found that dogs in contact with other animals had shorter relative telomere lengths than those with no contact. Although socialization is a well-established factor in the healthy development of dogs, social dynamics can pose additional challenges to these animals.

Animals kept in crowded environments may experience an increased likelihood of social confrontations and heightened stress responses, particularly when spatial restrictions and the inability to avoid other individuals are present, as group living can lead to greater competition for resources [45]. Studies conducted on mice and hyenas demonstrate that crowded groups and social hierarchies impact telomere attrition. Similarly, a study on African grey parrots found that social isolation resulted in shorter telomeres. These findings help illustrate the complexity of the relationship between social settings and telomere dynamics.

The relationships found in our results do not establish a clear parameter for types of interaction that may affect telomere length, as both dogs with only one to two contacts and those with five or more contacts had shorter telomeres. However, certain characteristics of the surveyed groups may help further understand what might underlie these associations. In our study, many dogs had contact with others; still, they were not necessarily housed together, or they may have been housed with individuals without the possibility of avoidance, such as in shared kennels. Furthermore, the presence of another individual does not necessarily equate to companionship, especially if dogs live in a mixed-species household. All these factors may contribute to the variation observed in the results.

As our questionnaire did not provide further detail beyond the number of animal contacts to better understand these findings, future studies could manipulate social settings more deliberately to explore how varying social environments impact telomere dynamics in dogs.

Previous studies have established a relationship between telomere shortening and dogs’ breed-predicted lifespan [10]; however, our study did not find any significant effects of age or breed on relative telomere length (rTL). This discrepancy could be attributed to the heterogeneous nature of our sample, which included a wide range of breeds, ages, and sizes. The variability in telomere lengths within the different breeds and age groups may have masked any potential relationships, highlighting the complexity of factors influencing rTL in dogs. Future research with a more homogenous sample or larger sample size could provide further insights into how age and breed specifically impact telomere dynamics.

Finally, despite differences in management and housing conditions, as well as the distribution of rTL, varying among locations, there was no significant difference between the two groups of police dogs (from Brazil and the UK). Only the type of work the dogs were engaged in showed a substantial correlation with telomere length.

Based on the type of work, dogs commonly used in street patrols, known as capture dogs, showed increased telomere length compared to other groups. Police dogs are generally trained for specific duties, such as security, patrol, or detection; sometimes, they are cross-trained for multiple purposes [44]. Overall, research in this area is still limited, but previous studies indicate that the nature of a dog’s work may increase stress responses and influence their reproductive performance and life quality [48].

Due to the inherent characteristics of the type of work, patrol dogs may work in less demanding conditions and be less exposed to risk than dogs in other groups, such as those involved in search and rescue and explosive detection [51]. Previous studies have shown that these dogs experience significant psychological and physiological stress due to frustration and high-intensity tasks, which can affect their performance and welfare [51,52]. Moreover, specific training protocols, such as exposure to harmful chemicals in drug detection training, could also potentially influence telomere dynamics, further highlighting the complex relationship between training-related stress and telomere attrition.

Training experience and social interactions with handlers may also play a role. Dogs with higher trainability and less exposure to acute stressors tend to maintain better telomere dynamics, as observed in studies linking behavioral factors to telomere length [37]. Studies dedicated to evaluating the stress dynamics between dogs and handlers have highlighted that older and experienced dogs [53] and also experienced handlers [54] can contribute to better performance and alleviate the stress of working demands.

Lastly, differences in stress responses are also found among dog breeds used in police work, influenced by genetic, physiological, and behavioral [55] factors, all factors that can independently or jointly influence cell aging. Common breeds used for patrolling and other breeds selected for cooperative tasks, such as German Shepherds, may handle stress better than independent worker breeds due to their genetic predisposition for focused attention and human interaction [56].

We propose that future research incorporate location-specific analyses to better understand how regional factors—such as job responsibilities, environmental stressors, and work intensity—affect telomere length in working dogs.

Overall, our study identified several background factors that may impact a dog’s telomere length; to our knowledge, no previous research involving non-human species has examined the influence of these multiple background factors on telomere attrition dynamics.

Our findings align with the framework proposed by [50], highlighting telomere attrition as a key hallmark of aging in domestic dogs. This study emphasizes that telomere shortening is critical to biological aging and is influenced by various environmental and behavioral factors. Specifically, our research found that housing conditions, exercise, and social interactions had a significant impact on dogs’ relative telomere length (rTL). These factors suggest that lifestyle choices can accelerate or delay aging through telomere attrition. This supports the notion that life experiences, such as sleeping sites and daily walks, are integral to understanding biological aging in dogs.

Despite our considerable sample size, which included dogs from diverse backgrounds, sexes, and age groups, we diluted the overall sample size within specific categories. This dilution may limit our ability to identify additional factors influencing relative telomere length. Moreover, another factor that could hinder comparisons among dogs is the significant number of variables that define a dog’s household environment. Therefore, future research should focus on uniform sampling to effectively identify factors contributing to premature aging in dogs, using methodologies similar to those employed in epidemiological studies of human aging and health [49]. Such an approach would enable the precise identification of environmental factors that contribute to biological aging, thus providing valuable insights into the dynamics of aging in non-human species.

## 5. Conclusions

Our findings contribute to a holistic perspective, advocating that environmental and behavioral experiences should be considered when evaluating canine health and welfare. This aligns with the suggestion that telomere length may serve as a valuable biomarker for assessing the impact of these factors on aging and overall welfare. In contrast to many animal welfare assessments that focus solely on one polarity, this approach provides a more comprehensive understanding of how various beneficial and harmful life experiences contribute to a dog’s overall well-being and aging process. By considering the full spectrum of experiences, we can better evaluate and address the needs of dogs throughout their lives.

Our results demonstrated that dogs from different backgrounds experience varying stressors throughout their lives, which can contribute to shorter telomere lengths. Interestingly, while individual characteristics such as age and breed may not directly lead to accelerated aging, dogs with similar traits living in comparable environments tend to show similar patterns of telomere shortening. Laboratory dogs were found to have the shortest relative telomere lengths among the groups studied, supporting the assumption that dogs in such conditions generally experience lower welfare than those in other conditions. On the other hand, police dogs used for patrolling and capture exhibit longer telomeres than their counterparts. Given the challenging nature of their work and the diverse roles that police dogs can undertake, understanding the impacts of various activities may play a vital role in their day-to-day activities and overall health and welfare.

The variation in telomere length among pet dogs was primarily associated with their sleeping arrangements, suggesting that housing conditions significantly elicit stress responses that could accelerate telomere attrition. Factors such as exercise and social interactions were also found to influence telomere length across different dog groups. However, further research is needed to understand the thresholds between these variables better—whether they help prevent telomere attrition or contribute to stress responses that may accelerate aging. Understanding these dynamics is crucial for improving the welfare and longevity of dogs.

While this study provides valuable insights into telomere length as a potential welfare biomarker, several limitations should be acknowledged. First, chronic stress was not directly assessed through additional physiological or behavioral markers, limiting our ability to correlate telomere attrition with specific stress responses. Second, the absence of inter-run calibrators (IRCs) in qPCR may introduce minor plate-to-plate variation, though standard curve normalization and technical replicates were employed to mitigate this. Third, factors such as dietary habits and social interactions, although recorded, were excluded from the final analysis due to data completeness constraints. Additionally, variation in housing conditions across groups, particularly among shelter and laboratory dogs, may have influenced stress exposure differently, and the impact of shelter duration remains an open question. Future studies should integrate complementary stress biomarkers, such as hair cortisol and oxidative stress indicators, alongside telomere length analysis to provide a more comprehensive assessment of canine welfare.

Early identification of accelerated aging is vital, as it helps detect health issues at an early stage, making treatment more straightforward, affordable, and likely more effective [57]. This strategy could prolong the service life of working dogs while safeguarding their well-being. As a result, the findings of this study may have significant economic benefits for both pet owners and organizations employing working dogs, potentially reducing veterinary costs, extending the active service period of the dogs, and enhancing their overall health and quality of life.

Despite the limitations in sample sizes across the groups, we believe this initial approach to evaluating the effect of the environment on relative telomere length holds significant potential as a tool for measuring animal welfare through aging. The findings suggest that the cumulative experiences of animals can influence biological aging, making telomere length a promising indicator. Further studies are needed to explore the relationship between life experiences and aging, providing a deeper understanding of how environmental and social factors contribute to animals’ overall health and well-being.

## Figures and Tables

**Table 1:** Summary of the characteristics of the different dog groups sampled for relative telomere length assessment.

Background	N	Males	Females	Age (±SD)
Pet	84	45	39	4.05 ± 3.42
Shelter	56	33	21	4.00 ± 2.25
Work (BR)	46	32	14	3.21 ± 2.21
Work (UK)	20	17	3	5.27 ± 4.58
Laboratory	21	8	13	3.50 ± 2.49
Rehomed	23	9	14	1.80 ± 1.33

**Table 2:** The mean relative telomere length (rTL) found for each age band (in years) assessed using qPCR from the samples of 250 dogs used in the study.

Age	N	Mean	StDev	Variance	CoefVar	Minimum	Median	Maximum	IQR
1	51	0.746	0.087	0.008	11.666	0.62	0.720	0.97	0.110
2	63	0.750	0.083	0.007	11.007	0.55	0.740	0.99	0.095
3	31	0.750	0.079	0.006	10.561	0.62	0.740	0.96	0.080
4	20	0.727	0.063	0.004	8.701	0.64	0.725	0.92	0.075
5	22	0.752	0.072	0.005	9.544	0.61	0.735	0.92	0.090
6	17	0.729	0.070	0.005	9.652	0.61	0.730	0.86	0.090
7	17	0.741	0.070	0.005	9.421	0.58	0.740	0.91	0.050
8	4	0.778	0.116	0.013	14.939	0.62	0.795	0.90	0.078
9	6	0.750	0.052	0.003	6.954	0.70	0.745	0.83	0.073
10	5	0.744	0.069	0.005	9.244	0.68	0.720	0.84	0.100
11	5	0.672	0.094	0.009	14.015	0.55	0.660	0.78	0.130
12	3	0.673	0.093	0.009	13.799	0.57	0.700	0.75	0.090
13	2	0.795	0.205	0.042	25.794	0.65	0.795	0.94	0.145
14	1	0.760	NA	NA	NA	0.76	0.760	0.76	0.000

IQR = interquartile range. N = Number of dogs per age group.

**Table 3:** GLM results for the optimal model describing the effect of group category, sex, sleep site, exercise, and interaction with other animals on the relative telomere length of domestic dogs ^1^.

Parameters	Estimate ± SD	t Value	*p*
Intercept	−0.1888 ± 0.0526	−3.588	0.0004 ***
Sex male ^a^	0.0270 ± 0.0124	2.170	0.0311 *
Sleep kennel outside ^b^	−0.0918 ± 0.0358	−2.565	0.0109 *
Sleep owner’s bed	0.0062 ± 0.0215	0.288	0.7732
Walk every other day ^c^	0.1351 ± 0.0289	4.683	<0.0001 ***
Walk not walked	0.0695 ± 0.0295	2.358	0.0192 *
Walk once a week	−0.0350 ± 0.0344	−1.016	0.3108
Contact 1–2 ^d^	−0.0635 ± 0.0232	−2.735	0.0067 **
Contact 3–4	−0.0392 ± 0.0244	−1.605	0.1098
Contact 5+	−0.0749 ± 0.0294	−2.545	0.0116 *
Category lab ^e^	−0.1737 ± 0.0448	−3.879	0.0001 ***
Category pet	−0.1277 ± 0.0426	−3.000	0.0030 **
Category rehomed	−0.0122 ± 0.0373	−0.326	0.7444
Category shelter	−0.0330 ± 0.0362	−0.911	0.3632
Category UK work	0.0529 ± 0.0309	1.711	0.0884

^1^ Best model fit AICc = −619.84. Non-significant (NS) variables were removed from the model. The remaining NS factors could not be discarded without compromising the model efficiency. ^a^ Sex: Female is the reference group. ^b^ Sleeps outside, inside, shares owner’s bed: Inside the house is the reference group. ^c^ Frequency of exercise—Walks every day, once a week, not walked, or every other day: Walks every day is the reference group. ^d^ Social interactions—No contact (kept alone), 1–2 animals, 3–4 animals, more than 5 animals: No contact is the reference group. ^e^ Background: Lab dogs, pet dogs, rehomed dogs, shelter dogs, UK working dogs, BR working dogs: BR work is the reference group. *** *p* ≤ 0.001, ** *p* ≤ 0.01, * *p* ≤ 0.05.

**Table 4:** Distribution of the mean relative telomere length (rTL) for 65 working dogs included in the study based on the type of work performed.

Work Type	N	Mean	StDev	Variance	Min	Median	Max	IQR	CoefVar
Breeder	2	0.705	0.092	0.008	0.64	0.705	0.77	0.065	13.039
Drug/gun	14	0.828	0.084	0.007	0.7	0.825	0.96	0.135	10.164
Education	6	0.707	0.052	0.003	0.64	0.695	0.79	0.047	7.362
Explosives	10	0.764	0.057	0.003	0.69	0.765	0.84	0.085	7.435
Retired	6	0.788	0.133	0.018	0.57	0.78	0.94	0.13	16.817
Search	7	0.693	0.042	0.002	0.64	0.69	0.77	0.035	6.107
Tracking	20	0.826	0.086	0.007	0.68	0.815	0.97	0.122	10.395

IQR = interquartile range. N = number of dogs per work type.

**Table 5:** GLM results for the optimal model examining the impact of group category, sex, and work type on the relative telomere length of working dogs ^1^.

Parameters	Estimate ± SD	t Value	*p*
Intercept	0.6400 ± 0.0784	8.156	<0.0001 ***
Group UK work^a^	−0.0511 ± 0.0262	−1.950	0.0561
Sex male ^c^	0.0381 ± 0.0234	1.627	0.1093
Working type: capture ^d^	0.1730 ± 0.0833	2.076	0.0425 *
Working type: drugs and guns	0.1606 ± 0.0829	1.936	0.0579
Working type: educational	0.0412 ± 0.0861	0.478	0.6343
Working type: explosives	0.1220 ± 0.0850	1.435	0.1569
Working type: retired	0.1506 ± 0.0883	1.704	0.0939
Working type: search and rescue	0.0342 ± 0.0855	0.400	0.6905

^1^ Best models for relative telomere length AICc = −644.40. Non-significant (NS) variables were removed from the model. The remaining NS factors could not be discarded without compromising the model efficiency. ^a^ UK work or BR work: BR work is the reference group. ^c^ Sex: Female is the reference group. ^d^ Type of work: Breeder, capture, drugs and guns, educational, explosives, retired, search and rescue: Breeder is the reference group. * *p* ≤ 0.05, *** *p* ≤ 0.0001.

## Data Availability

The dataset is available upon request from the authors.

## Supplementary Materials

The following supporting information can be downloaded at https://www.mdpi.com/article/10.3390/vetsci12050491/s1, Table S1: Information from the 250 dogs (*Canis familiaris*) sampled for relative telomere length (rTL) analysis used in the current study; Table S2: Information regarding the background of the dogs (*Canis familiaris*) that were sampled for this study to explore the impact of stress, sociality, and exercise on dogs’ cellular aging; and Table S3: Categories of police working dogs, as grouped for exploring the impact of stress, sociality, and exercise on dogs’ cellular aging. Figure S1: Informed consent statement.
