# Supplementary material for: Telomere Tales: Exploring the Impact of Stress, Sociality, and Exercise on Dogs’ Cellular Aging
Source: Vet Sci. 2025 May 19;12(5):491. doi: 10.3390/vetsci12050491 (PMC12115685; doi:10.3390/vetsci12050491)
Supplement: Supplementary file 1 [file vetsci-12-00491-s001.zip › vetsci-3416408-supplementary.pdf]

Table S1. Information from the 264 dogs (*Canis familiaris*) sampled for relative telomere length (rTL) analysis used in the current study.

Sample	Category	ID	Sex	Age	Breed	Breed Group
1	Lab	OPS1A	2	6	Mix	Mix
2	Lab	OPS2A	2	6	Mix	Mix
3	Lab	OPS3A	2	1	Mix	Mix
4	Lab	OPS4A	2	1	Mix	Mix
5	Lab	OPS5A	2	6	Mix	Mix
6	Lab	OPS6A	2	1	Mix	Mix
7	Lab	OPS7A	2	1	Mix	Mix
8	Lab	OPS8A	2	1	Mix	Mix
9	Lab	OPS9A	2	3	Mix	Mix
10	Lab	OPS10A	2	5	Mix	Mix
11	Lab	OPS11A	2	6	Mix	Mix
12	Lab	OPS12A	2	8	Mix	Mix
13	Lab	OPS13A	2	6	Mix	Mix
14	Lab	OPS14A	1	2	Mix	Mix
15	Lab	OPS15A	1	2	Mix	Mix
16	Lab	OPS16A	1	2	Mix	Mix
17	Lab	OPS17A	1	2	Mix	Mix
18	Lab	OPS18A	1	6	Mix	Mix
19	Lab	OPS19A	1	6	Mix	Mix
20	Lab	OPS20A	1	0.5	Mix	Mix
21	Lab	OPS21A	1	2	Mix	Mix
22	Pet	FPS1	1	1.25	Poodle mix	Mix
23	Pet	FPS2	2	7	Jack russel	Terrier
24	Pet	FPS3	2	1	Chihuahua	Toy
25	Pet	FPS4	2	9	Pug	Toy
26	Pet	FPS5	1	1.08	Black russian terrier	Working
27	Pet	FPS6	2	10	Jack russel	Terrier
28	Pet	FPS7	2	0.58	Chow chow	Utility
29	Pet	FPS8	2	0.41	Schnauzer	Utility
30	Pet	FPS9	1	1	Poodle	Utility
31	Pet	FPS10	2	0.5	Yorky mix	Mix
32	Pet	FPS11	2	3	Spring spaniel	Gundog
33	Pet	FPS12	2	0.25	Dashund	Hound
34	Pet	FPS13	2	9	West highlander terrier	Terrier
35	Pet	FPS14	1	3	Mix	Mix
36	Pet	FPS15	2	1.33	Spring spaniel	Gundog
37	Pet	FPS16	1	3	Mix	Mix
38	Pet	FPS17	1	1.25	Pug	Toy
39	Pet	FPS18	1	4	Chihuahua mix	Toy
40	Pet	FPS19	1	4	Greyhound	Hound
41	Pet	FPS20	2	2	Border collie	Pastoral
42	Pet	FPS21	1	7	Mixed breed	Mix

43	Pet	FPS22	2	4	Poodle	Utility
44	Pet	FPS23	2	7	Mix	Mix
45	Pet	FPS24	2	1.5	Greyhound	Hound
46	Pet	FPS25	2	4	Border collie	Pastoral
47	Pet	FPS26	2	6	Mix	Mix
48	Pet	FPS27	2	7	Spring spaniel	Gundog
49	Pet	FPS28	2	2	Shih tzu	Utility
50	Pet	FPS29	1	4	Australian Shepard	Pastoral
51	Pet	FPS30	1	7	Mix	Mix
52	Pet	FPS31	1	0.5	Poodle	Utility
53	Pet	FPS32	1	4	Lhasa Apso	Utility
54	Pet	FPS33	1	5	Collie	Pastoral
55	Pet	FPS34	1	4	Dashund	Hound
56	Pet	FPS35	1	4	Chihuahua	Toy
57	Pet	FPS36	1	1	Chihuahua	Toy
58	Pet	FPS37	1	0.66	Bichon frise	Toy
59	Pet	FPS38	1	11	Jack Russel	Terrier
60	Pet	FPS39	1	0.88	Labradoodle	Mix
61	Pet	FPS40	1	2	Dogue de Bordeaux	Working
62	Pet	FPS41	1	0.58	White German Shepard	Pastoral
63	Pet	FPS42	1	0.33	Pinscher Toy Poodle	Toy
64	Pet	FPS43	1	1	Chihuahua Daschund	Toy
65	Pet	FPS44	2	0.33	Basset hound	Hound
66	Pet	FPS45	2	11	Cocker Spaniel	Gundog
67	Pet	FPS46	1	3	Bedlington terrier	Terrier
68	Pet	FPS47	2	7	Sharpei	Utility
69	Pet	FPS48	2	10.5	Spring spaniel	Gundog
70	Pet	FPS49	1	1.5	Cocker Spaniel	Gundog
71	Pet	FPS50	1	1.3	Mix	Mix
72	Pet	FPS51	1	12	Shih tzu	Utility
73	Pet	FPS52	2	2	Pug	Toy
74	Pet	FPS53	2	1	Jack russel chihuahua	Toy
75	Pet	FPS54	1	3	Mix	Mix
76	Pet	FPS55	2	2	Sprocker Spaniel	Gundog
77	Pet	FPS56	1	2	Poodle	Utility
78	Pet	FPS57	2	2	Chihuahua	Toy
79	Pet	FPS58	2	11	Mix	Mix
80	Pet	FPS59	1	7	German Spitz	Utility
81	Pet	FPS60	1	0.5	Goldendoodle	Mix
82	Pet	FPS61	1	0.75	Goldendoodle	Mix
83	Pet	FPS62	1	0.75	Goldendoodle	Mix
84	Pet	FPS63	2	9.5	Border collie	Pastoral
85	Pet	FPS64	1	0.5	Labrador	Gundog
86	Pet	FPS65	2	2	Spaniel	Gundog
87	Pet	FPS66	1	3	Spaniel	Gundog

88	Pet	FPS67	1	7	Yorkshire	Toy
89	Pet	FPS68	1	1	Yorkshire	Toy
90	Pet	FPS69	2	11	Yorkshire	Toy
91	Pet	FPS70	1	2	Yorkshire	Toy
92	Pet	FPS71	2	6	Yorkshire	Toy
93	Pet	FPS72	2	5	Yorkshire	Toy
94	Pet	FPS73	2	5	Yorkshire	Toy
95	Pet	FPS74	2	5	Yorkshire	Toy
96	Pet	FPS75	2	1	Yorkshire	Toy
97	Pet	FPS76	2	6	Mix	Mix
98	Pet	FPS77	2	5	Poodle	Utility
99	Pet	FPS78	1	4	Kuvacz	Pastoral
100	Pet	FPS79	1	1.7	Shih tzu	Utility
101	Pet	FPS80	1	10	Yorkshire	Toy
102	Pet	FPS81	1	3	Yorkshire	Toy
103	Pet	FPS83	1	8	Shih tzu	Utility
104	Pet	FPS84	1	6	Shih tzu	Utility
105	Pet	FPS85	2	13	Mix	Mix
106	Rehome	WKDS1	1	1	Spring spaniel	Gundog
107	Rehome	WKDS2	1	1.5	Labrador	Gundog
108	Rehome	WKDS3	2	1	Labrador	Gundog
109	Rehome	WKDS4	2	1.25	Labrador	Gundog
110	Rehome	WKDS5	2	1.6	German Pointer	Gundog
111	Rehome	WKDS6	2	2	Mixed	Gundog
112	Rehome	WKDS7	2	0.8	Labrador	Gundog
113	Rehome	WKDS8	1	1.25	Spring spaniel	Gundog
114	Rehome	WKDS9	2	2	Golden Retriver	Gundog
115	Rehome	WKDS10	1	2	German Pointer	Gundog
116	Rehome	WKDS11	2	5	Spring spaniel	Gundog
117	Rehome	WKDS12	2	2.1	Spring spaniel	Gundog
118	Rehome	WKDS13	2	1	Spring spaniel	Gundog
119	Rehome	WKDS14	2	2	Spring spaniel	Gundog
120	Rehome	WKDS15	1	1.5	Labrador	Gundog
121	Rehome	WKDS16	1	0.83	Spring spaniel	Gundog
122	Rehome	WKDS17	1	1	Spring spaniel	Gundog
123	Rehome	WKDS18	2	1	Spring spaniel	Gundog
124	Rehome	WKDS19	1	0.66	English Setter	Gundog
125	Rehome	WKDS20	1	0.75	German Pointer	Gundog
126	Rehome	WKDS21	2	5	Spring spaniel	Gundog
127	Rehome	WKDS22	2	2	Golden Retriver	Gundog
128	Rehome	WKDS23	2	5	Labrador	Gundog
129	Shelter	SS1	2	5	Mix	Mix
130	Shelter	SS2	1	2	Mix	Mix
131	Shelter	SS4	2	5	Mix	Mix
132	Shelter	SS5	1	2	Mix	Mix

133	Shelter	SS6	2	2	Mix	Mix
134	Shelter	SS7	1	2	Mix	Mix
135	Shelter	SS8	1	2	Mix	Mix
136	Shelter	SS9	1	9	Mix	Mix
137	Shelter	SS10	1	2	Mix	Mix
138	Shelter	SS11	1	3	Mix	Mix
139	Shelter	SS12	2	2	Mix	Mix
140	Shelter	SS13	1	5	Mix	Mix
141	Shelter	SS14	2	2	Mix	Mix
142	Shelter	SS15	1	9	Mix	Mix
143	Shelter	SS17	1	10	Mix	Mix
144	Shelter	SS18	1	2	Mix	Mix
145	Shelter	SS19	1	4	Mix	Mix
146	Shelter	SS20	1	7	Mix	Mix
147	Shelter	SS21	2	7	Mix	Mix
148	Shelter	SS22	1	4	Mix	Mix
149	Shelter	SS23	2	3	Mix	Mix
150	Shelter	SS24	1	5	Mix	Mix
151	Shelter	SS25	2	5	Mix	Mix
152	Shelter	SS26	2	3	Mix	Mix
153	Shelter	SS27	2	3	Mix	Mix
154	Shelter	SS28	1	2	Mix	Mix
155	Shelter	SS29	1	6	Mix	Mix
156	Shelter	SS30	1	4	Mix	Mix
157	Shelter	SS31	2	6	Mix	Mix
158	Shelter	SS32	2	4	Mix	Mix
159	Shelter	SS33	1	7	Mix	Mix
160	Shelter	SS34	2	3	Mix	Mix
161	Shelter	SS35	1	3	Mix	Mix
162	Shelter	SS36	2	3	Mix	Mix
163	Shelter	SS37	1	4	Mix	Mix
164	Shelter	SS38	1	5	Mix	Mix
165	Shelter	SS39	1	3	Mix	Mix
166	Shelter	SS40	1	2	Mix	Mix
167	Shelter	SS41	1	2	Mix	Mix
168	Shelter	SS42	1	2	Mix	Mix
169	Shelter	SS43	2	2	Mix	Mix
170	Shelter	SS44	2	2	Mix	Mix
171	Shelter	SS45	1	2	Mix	Mix
172	Shelter	SS46	1	2	Mix	Mix
173	Shelter	SS47	2	5	Mix	Mix
174	Shelter	SS48	1	3	Mix	Mix
175	Shelter	SS49	1	3	Mix	Mix
176	Shelter	SS50	1	9	Mix	Mix
177	Shelter	SS51	2	2	Mix	Mix

178	Shelter	SS52	1	3	Mix	Mix
179	Shelter	SS53	2	9	Mix	Mix
180	Shelter	SS54	2	6	Mix	Mix
181	Shelter	SS55	2	2	Mix	Mix
182	Shelter	SS56	1	5	Mix	Mix
183	UK Work	MPS1	1	0.75	German Shepard	Pastoral
184	UK Work	MPS2	1	0.75	German Shepard	Pastoral
185	UK Work	MPS3	1	7	Spring Spaniel	Gundog
186	UK Work	MPS4	1	4	Spring Spaniel	Gundog
187	UK Work	MPS5	1	1	German Shepard	Pastoral
188	UK Work	MPS6	1	13	Spring Spaniel	Gundog
189	UK Work	MPS7	1	1	German Shepard	Pastoral
190	UK Work	MPS8	1	2	Spring Spaniel	Gundog
191	UK Work	MPS9	1	10	Spring Spaniel	Gundog
192	UK Work	MPS10	1	3	German Shepard	Pastoral
193	UK Work	MPS11	1	12	Spring Spaniel	Gundog
194	UK Work	MPS12	1	12	Spring Spaniel	Gundog
195	UK Work	MPS13	2	1	Spring Spaniel	Gundog
196	UK Work	MPS14	1	7	German Shepard	Pastoral
197	UK Work	MPS15	1	14	German Shepard	Pastoral
198	UK Work	MPS16	1	6	German Shepard	Pastoral
199	UK Work	MPS17	1	3	German Shepard	Pastoral
200	UK Work	MPS18	2	4	German Shepard	Pastoral
201	UK Work	MPS19	2	3	Spring Spaniel	Gundog
202	UK Work	MPS20	1	1	Spring Spaniel	Gundog
203	BR Work	WS1	1	8	German Shepard	Pastoral
204	BR Work	WS2	2	8	German Shepard	Pastoral
205	BR Work	WS3	1	2.6	Border collie	Pastoral
206	BR Work	WS4	1	2.6	Border collie	Pastoral
207	BR Work	WS5	2	1.3	Malinois	Pastoral
208	BR Work	WS6	1	4	Malinois	Pastoral
209	BR Work	WS7	2	0.6	Malinois	Pastoral
210	BR Work	WS8	2	0.6	Malinois	Pastoral
211	BR Work	WS9	1	0.3	Border collie	Pastoral
212	BR Work	WS10	1	4	German Shepard	Pastoral
213	BR Work	WS11	2	1	German Shepard	Pastoral
214	BR Work	WS12	2	1	German Shepard	Pastoral
215	BR Work	WS13	1	3	German Shepard	Pastoral
216	BR Work	WS14	1	0.7	Rottweiler	Working
217	BR Work	PMS1	2	3	Malinois	Pastoral
218	BR Work	PMS2	1	7	Labrador	Gundog
219	BR Work	PMS3	1	5	German Shepard	Pastoral
220	BR Work	PMS4	2	7	German Shepard	Pastoral
221	BR Work	PMS5	2	6	Malinois	Pastoral
222	BR Work	PMS7	2	5	German Shepard	Pastoral

223	BR Work	PMS9	2	5	German Shepard	Pastoral
224	BR Work	PMS10	1	2	German Shepard	Pastoral
225	BR Work	PMS11	1	3	Malinois	Pastoral
226	BR Work	PMS12	1	2	Malinois	Pastoral
227	BR Work	PMS13	1	7	German Shepard	Pastoral
228	BR Work	PMS14	2	2	German Shepard	Pastoral
229	BR Work	PMS15	1	2	German Shepard	Pastoral
230	BR Work	PMS16	1	5	Malinois	Pastoral
231	BR Work	PMS17	2	2	Dutch Shepherd	Pastoral
232	BR Work	PMS18	1	3	Malinois	Pastoral
233	BR Work	PMS19	1	3	German Shepard	Pastoral
234	BR Work	PMS20	1	2	Labrador	Gundog
235	BR Work	PMS21	1	1	Malinois	Pastoral
236	BR Work	PMS22	1	1	Malinois	Pastoral
237	BR Work	PMS23	1	1	Malinois	Pastoral
238	BR Work	PMS24	1	1	German Shepard	Pastoral
239	BR Work	PMS25	1	1	Malinois	Pastoral
240	BR Work	PMS26	1	4	Malinois	Pastoral
241	BR Work	PMS27	1	1	Malinois	Pastoral
242	BR Work	PMS28	1	5	Malinois	Pastoral
243	BR Work	PMS29	2	2	Malinois	Pastoral
244	BR Work	PMS30	1	2	German Shepard	Pastoral
245	BR Work	PMS31	1	7	Labrador	Gundog
246	BR Work	PMS32	1	3	Malinois	Pastoral
247	BR Work	PMS33	1	4	Dutch Shepherd	Pastoral
248	BR Work	PMS34	1	6	German Shepard	Pastoral

Table S2. Information regarding the background of the dogs (*Canis familiaris*) that were sampled for this study to explore the impact of Stress, Sociality, and Exercise on Dogs' Cellular Aging

Sex	Age	Breed/Breed Group	Origin	Neutered	Healthy	Training	Treat	Sleep	Walk	People Contact	Animal Contact
1 Male	Years	1 Gundog	1 First owner	1 No	1 Yes	1 No	1 No	1 Kennel outside	1 Everyday	1 One to two people	1 No contact
2 Female		2 Hound	2 Adopted	2 Yes	2 No	2 Yes	2 Yes	2 Inside house	2 Once a week	2 Between 3-4 people	2 One to two animals
		3 Pastoral	3 Laboratory	3 Vasectomy				3 Owners bed	3 Don't walk	3 More than 5 people	3 Between 3-4 animals
		4 Terrier							4 Every other day		4 More than 5 animals
		5 Toy									
		6 Utility									
		7 Working									
		8 Mix									

Table S3. Classification and Description of Working Dog Types by Role and Geographic Origin

Working Type	Description	Country of Origin
1. Drugs, Guns	Dogs trained for detection of illegal drugs or firearms, often working with law enforcement in controlled settings.	UK and Brazil working dogs
2. Tracking	Dogs used for tracking suspects or individuals based on scent during patrol, often involved in law enforcement activities.	Brazil working dogs
3. Breeder	Dogs bred for specific working purposes but not yet fully trained or employed in a professional capacity. These dogs may be involved in breeding programs for future working dogs.	Brazil working dogs
4. Educational Dog	Dogs used in training programs to teach basic obedience, often involved in educational settings or assisting with training other dogs. These dogs might not have a defined specific working role yet.	Brazil working dogs
5. Explosives	Dogs trained to detect explosives in various environments such as airports, security checks, or public events.	UK and Brazil working dogs
6. Search Dog	Dogs specifically trained for search and rescue operations, including locating missing people, tracking or sniffing for signs of life or scent in disaster areas.	Brazil working dogs
7. Retired	Dogs that have previously been employed in one of the above working roles but are no longer active due to age, health, or retirement from active duty.	UK and Brazil working dogs

Ethical Approval Reference Number: STR1617-22

Name of Researcher: Luisa Dutra

I confirm that I have read and understood the information sheet for the above study (version 1: 01/02/16) and understand what my dog's, and my, contribution will be	Yes	No
I have been given the opportunity to ask questions (face to face, via telephone and/or e-mail)	Yes	No
I agree to have my dog photographed during the study.	Yes	No
I agree to have my dog's mouth swab sampled during the study.	Yes	No
I understand that my dog's, and my, participation is voluntary and that I can withdraw my dog from the research at any time without giving any reason.	Yes	No
I understand how the researcher will use my dog's samples, who will see them, and how the data will be stored.	Yes	No
**I agree to my dog taking part in the above study**	Yes	No

Name of participant..

Signature...

Date..

Name of researcher taking consent.................................

Figure S1. Participants Consent Form
